# Ecological Theory in Early Literacy Assessment: A Preschool Teacher-Focused Scale (ETELAS-PT)

**DOI:** 10.3390/bs16101777

**Published:** 2026-09-29

**Authors:** Sırma Seda Bapoğlu Dümenci, Figen Turan

**Affiliations:** 1 Department of Child Development, Faculty of Health Sciences, Tarsus University, 33400 Mersin, Turkey; 2 Department of Child Development, Faculty of Health Sciences, Hacettepe University, 06100 Ankara, Turkey; fturan@hacettepe.edu.tr

**Keywords:** early literacy, Bronfenbrenner’s ecological theory, early literacy assessment, preschool teachers, scale development

## Abstract

Early literacy development in preschool is shaped by children’s individual skills and environmental contexts, including classroom practices, family routines, peer relationships, and cultural influences. While children’s engagement with literacy is embedded in daily interactions, most assessment tools remain focused on child outcomes, providing limited information about teachers’ practices and their engagement with the ecological theory that supports early literacy development. Based on Bronfenbrenner’s Ecological Theory, this study developed the Ecological Theory Early Literacy Assessment Scale (ETELAS-PT) to assess preschool teachers’ practices and awareness related to supporting children’s early literacy development within an ecological framework. Scale development involved a literature review, analysis of existing early literacy assessment instruments, and teacher interviews. The items were refined through expert review and pilot testing with nine teachers. The resulting instrument was administered to 221 preschool teachers (96.8% female; 56.9% with ≥10 years of experience) in public and private institutions. Psychometric analyses revealed a four-factor structure: Mesosystem, Exosystem, Macrosystem, and Chronosystem, with 25 items. Factor analysis showed high sampling adequacy (KMO = 0.894) and significant sphericity (χ^2^(300) = 3727.006, *p* < 0.001), with the four-factor structure explaining 64.35% of the total variance. Confirmatory analysis demonstrated an acceptable model fit (CMIN/df = 2.241; RMSEA = 0.069; SRMR = 0.075; CFI = 0.909; IFI = 0.910). The scale showed high internal consistency (α = 0.923; ω = 0.932). By identifying the ecological dimensions important to both early literacy development and preschool teachers’ practices and awareness, the ETELAS-PT provides a psychometrically supported tool that could be used for professional support as well as contextualized approaches towards early literacy practice in preschool.

## 1. Introduction

Early literacy, encompassing the knowledge, skills, and attitudes required before formal reading and writing (Kargın et al., 2015), is shaped by children’s ecological contexts. While individual skills, such as letter knowledge, are crucial, preschool teachers have observed that children’s engagement with print and language is influenced by factors, including family practices and peer interactions beyond the classroom. Despite this complexity and the theoretical lens of Bronfenbrenner’s Ecological Theory (Jaeger, 2016), which views literacy development as a dynamic process within multiple interacting systems, current early literacy assessment tools focus mainly on child-level outcomes. This narrow focus provides limited insight into the ecological contexts that are critical for a comprehensive understanding and intervention. Consequently, there is a gap in practical assessment instruments that enable preschool teachers to evaluate early literacy within a holistic ecological framework. Bronfenbrenner’s theory provides a framework for defining literacy development. It explains literacy experiences and shows how developing readers’ characteristics interact with proximal and distal influences to form their literacy ecologies (Bronfenbrenner, 1994). Preschool education is an important factor affecting early literacy (Campbell & Ramey, 1995). As Ergül et al. (2015) noted, although practices can be integrated into preschool activities to promote early literacy, preschool teachers lack the knowledge to support early literacy skills.

### 1.1. Ecological Approach to Early Literacy

The ecological approach to early literacy emphasizes environmental, social, and cultural contexts of literacy development, considering family, community, and cultural influences. This approach views development as a dynamic process influenced by the child’s ecological system. In line with this view, literacy growth is progressively recognized as a contextually embedded process in which the child’s own traits occur in relation to instructional methods, family contexts, socioeconomic situations, and additional sociocultural influences. More recent studies add further evidence, suggesting that results for literacy-related outcomes cannot be understood based on individual skills alone because children are exposed to various literacy experiences and development through interactions with teachers and in their wider environment (Palmqvist et al., 2025; Yang et al., 2026). According to (Bronfenbrenner, 1979), human development involves the interaction between individuals and their immediate settings, affected by relations between settings and larger contexts. The ecological environment consists of micro-, meso-, exo-, and macrosystems. A microsystem comprises activities, roles, and interpersonal relations in settings where people interact directly. The family, school, and peer groups are microsystems. A mesosystem includes interrelations among settings where the person participates, such as between home and school (Bronfenbrenner, 1979). An exosystem refers to settings not directly involving the person but affecting their environment, like a parent’s workplace. The macrosystem refers to consistencies in lower-order systems at the cultural level. Recent research introduced chronosystems, moving beyond chronological age (Bronfenbrenner, 1986). This theory explains how individual characteristics interact with influences to shape literacy ecology. Literacy interactions function as proximal processes, connecting readers with teachers, peers, parents, and materials within environments. Teachers are particularly important within these proximal contexts because their literacy-related beliefs and pedagogical orientations are reflected in the ways literacy experiences are organized and supported in early childhood settings (Matsumoto & Tsuneda, 2018). While Vygotsky’s zone of proximal development is similar, Bronfenbrenner emphasizes that all interactions are reciprocal, with elements facilitating change (Jaeger, 2016).

This ecological perspective is present in modern-day early childhood education frameworks. The Victorian Early Years Learning and Development Framework (VEYLDF), for example, is explicitly informed by an ecological framework, acknowledging that children learn and develop within their relationships with families, communities, cultures and contexts across time (State of Victoria, Department of Education, 2026). This framework provides an example of how ecological thinking can be engaged in the practice of contemporary early childhood education. Consistently, the American Academy of Pediatrics has also emphasized that early literacy development is considered to be influenced by environmental and relational factors such as the home literacy environment, quality of parent–child interactions, socioeconomic conditions, and access to community resources. Moreover, its recent policy statement emphasizes an eco-bio-developmental model in relation to emergent literacy (Klass et al., 2024).

**Figure 1:**
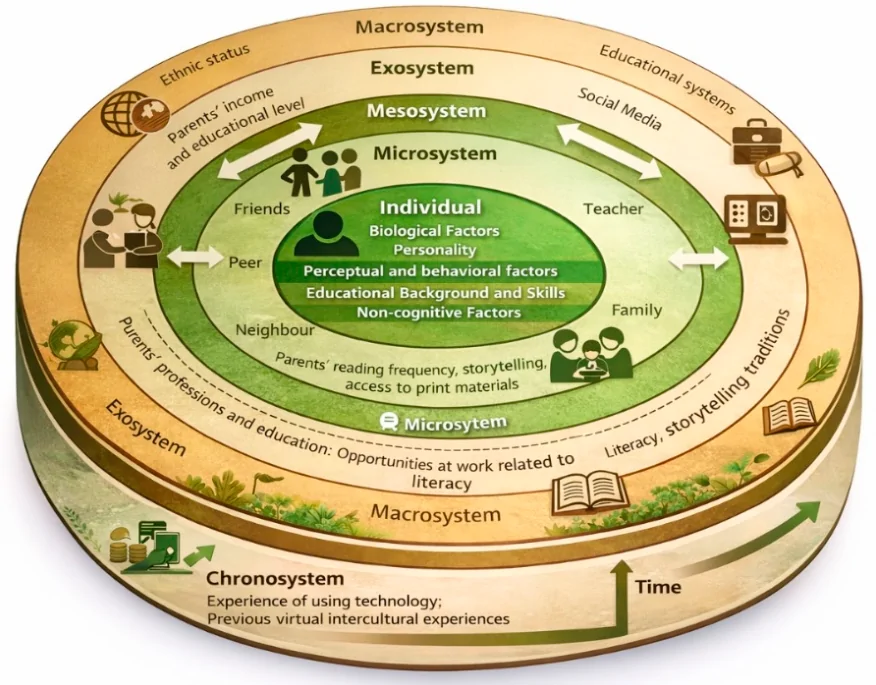
The ecological approach to early literacy.

As illustrated in Figure 1, the ecological approach points to a broader and more holistic perspective in understanding children’s development. This perspective encompasses both internal characteristics of the child and external environmental influences.

### 1.2. The Necessity of Early Literacy in Preschool Education

Studies highlight the predictive power of oral language and early literacy skills during preschool for later reading comprehension (National Early Literacy Panel, 2008; Conica et al., 2023). Recent intervention research further demonstrates that targeted language and literacy support during the preschool years can significantly improve foundational skills such as phonological awareness and lexical comprehension, reinforcing the importance of early identification and support (Majorano et al., 2022). Teacher Rating Scales of Early Academic Competence (TRS-EAC) assess academic abilities of children aged 36–72 months. Early literacy development begins at birth and develops over time (Senol & Turan, 2019). Assessment methods focusing on individual skills may not capture broader contextual factors. Classroom environments rich in literacy materials support this development (Nixon & Topping, 2001; Wayne et al., 2007). Significantly, the quality of instruction for early literacy is also contingent upon who teaches it and where it occurs. Recent findings show that professional development can develop educators’ practices to promote language and literacy acquisition in preschoolers, suggesting teachers are a key element of the context for early literacy (Chaitow et al., 2023). Preschool teachers must understand effective language instruction and assessment practices (Wilcox et al., 2020). A key challenge is preschool teachers’ limited knowledge about comprehensive early literacy support (Manning et al., 2017). Current assessments often focus narrowly on pre-reading skills while neglecting writing and conceptual development (Cviko et al., 2015; McKenney & Bradley, 2016). This is particularly important because early literacy development encompasses multiple interrelated skills, and early differences in language and literacy development may have implications for later reading trajectories, especially among children experiencing socioeconomic or other developmental risks (Bleses et al., 2021). This leads to unstable predictions of academic achievement, requiring comprehensive assessments (Pearson & Hiebert, 2010). Assessment tools must identify at-risk students and explain underlying reasoning (Bailey & Drummond, 2006). Bronfenbrenner’s Ecological Theory provides a framework for describing literacy development through individual characteristics and environmental influences (Baker, 2000; Jaeger, 2016). Accordingly, assessment approaches that consider not only children’s individual literacy skills but also the educational and contextual conditions in which those skills are supported may provide a more comprehensive understanding of early literacy development.

### 1.3. What Is the Ecological Theory-Based Early Literacy Assessment Scale?

Given the necessity for a holistic approach to early literacy assessment, this study introduces the ETELAS-PT to provide a teacher-focused ecological perspective on early literacy support. The scale is structured around four dimensions derived from Bronfenbrenner’s ecological systems theory:

Mesosystem: This level deals with the interaction between two or more different environmental contexts that impact children’s development, such as a collaboration between school and family, communication between teachers and parents, and relationships with other key stakeholders in education. It examines how teachers form such partnerships and how these interactions help children develop the literacy skills they need to achieve school readiness.

Exosystem: The broad national-level environment, which includes education in schools, news media, digital engines, and resources of learning beyond the direct reach of the child.

Macrosystem: Covers wider social, cultural and economic contexts, which include culturally informed understandings of abilities or practices, broader societal knowledge and experiences and other contextually relevant influences.

Chronosystem: This deals with the elements associated with change (over time) in different individuals and facilities. It consists of teachers being aware of early literacy development, tracking kids, communication with families, and how you guide families in supporting just eight endorsements over the years.

Specific skills and many factors in the environment (home, school and more broadly) all contribute to children’s literacy development (Weigel et al., 2005). While valid assessment tools allow identifying children at risk of developing early literacy difficulties who can benefit from focused intervention (Wildová & Kropáčková, 2015), it is also important to know the contexts where learning support takes place. Two or more sources of assessment methods that integrate classroom information, observations, and assessment data are commonly applied to enhance the basis for educational planning (Phillips et al., 2009). This more comprehensive view refers to ETELAS-PT, focusing specifically on preschool teachers’ ecological contexts, practices and observations in early literacy. The scale is intended not as a tool for identifying literacy difficulties in children, but rather as a focus for teachers reflecting on how their practices respond to educational, familial, cultural and other contextual factors related to early literacy development.

Accordingly, this study aimed to develop a valid and reliable instrument for assessing preschool teachers’ practices and awareness related to supporting early literacy development within the framework of Bronfenbrenner’s Ecological Theory. The study addressed the following research questions:

1. What factor structure emerges for the ETELAS-PT?
2. Does the proposed factor structure of the ETELAS-PT demonstrate an acceptable model fit?
3. Does the ETELAS-PT demonstrate adequate internal-consistency reliability?

## 2. Methods

This was a scale development study. The stages involved in the development of the ecological theory-based early literacy assessment scale ETELAS-PT were as follows:

### 2.1. The Development of the Ecological Theory-Based Early Literacy Assessment Scale-Teacher Form ETELAS-PT

**Figure 2:**
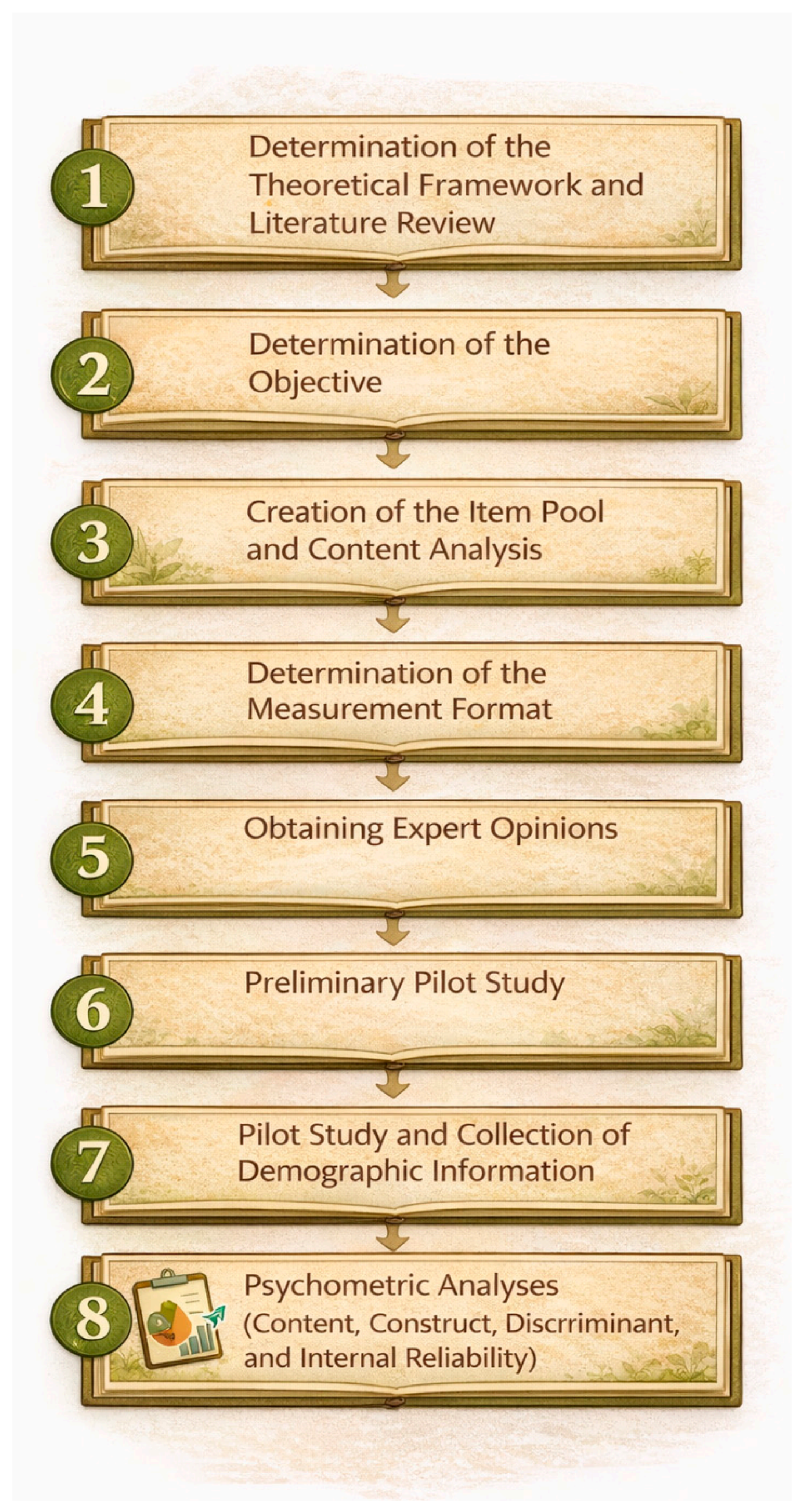
The scale development process consisted of the following stages.

As shown in Figure 2, the process consisted of a literature review and theoretical framework, expert assessment and observation, item pool creation, expert opinion, preliminary pilot study, pilot study, content validity, construct validity, discriminant external validity, and internal reliability stages. Literature review and theoretical framework: It is necessary to determine the conceptual and theoretical definitions of the characteristics to be measured, examine the structure of the variable to be measured, and conduct a literature review concerning the theoretical structure with which this variable is associated (Cohen & Swerdlik, 2018). Bronfenbrenner’s Ecological Theory was used (Bronfenbrenner, 1986). Similar scale items from the Early Literacy Knowledge Test (Laçin, 2020), the Interaction, Communication, and Literacy Skills Audit (ICL Skills Audit) (El-Choueifati et al., 2014), and the Emergent Literacy and Language Early Childhood Checklist for Teachers (ELLECCT) (Weadman et al., 2022) were examined.

Defining the objective: This was done specifically to develop a tool to evaluate preschool teachers in terms of their practices and beliefs regarding the promotion of early literacy development in children, within the context of Bronfenbrenner’s Ecological Theory. Instead of measuring teachers’ own early literacy skills or children’s performance in areas such as oral language, the instrument assesses teacher practices and knowledge across relevant ecological contexts related to early literacy.

Item pool creation: Items for the scale were obtained through methods such as reviewing similar tests, observation, and interviews.

Content analysis: This approach involves categorizing content to interpret qualitative data, enabling researchers to draw inferences about the communication context and purpose. It is widely used in social sciences, media studies, and psychology to analyze documents, speeches, interviews, and communications.

**Figure 3:**
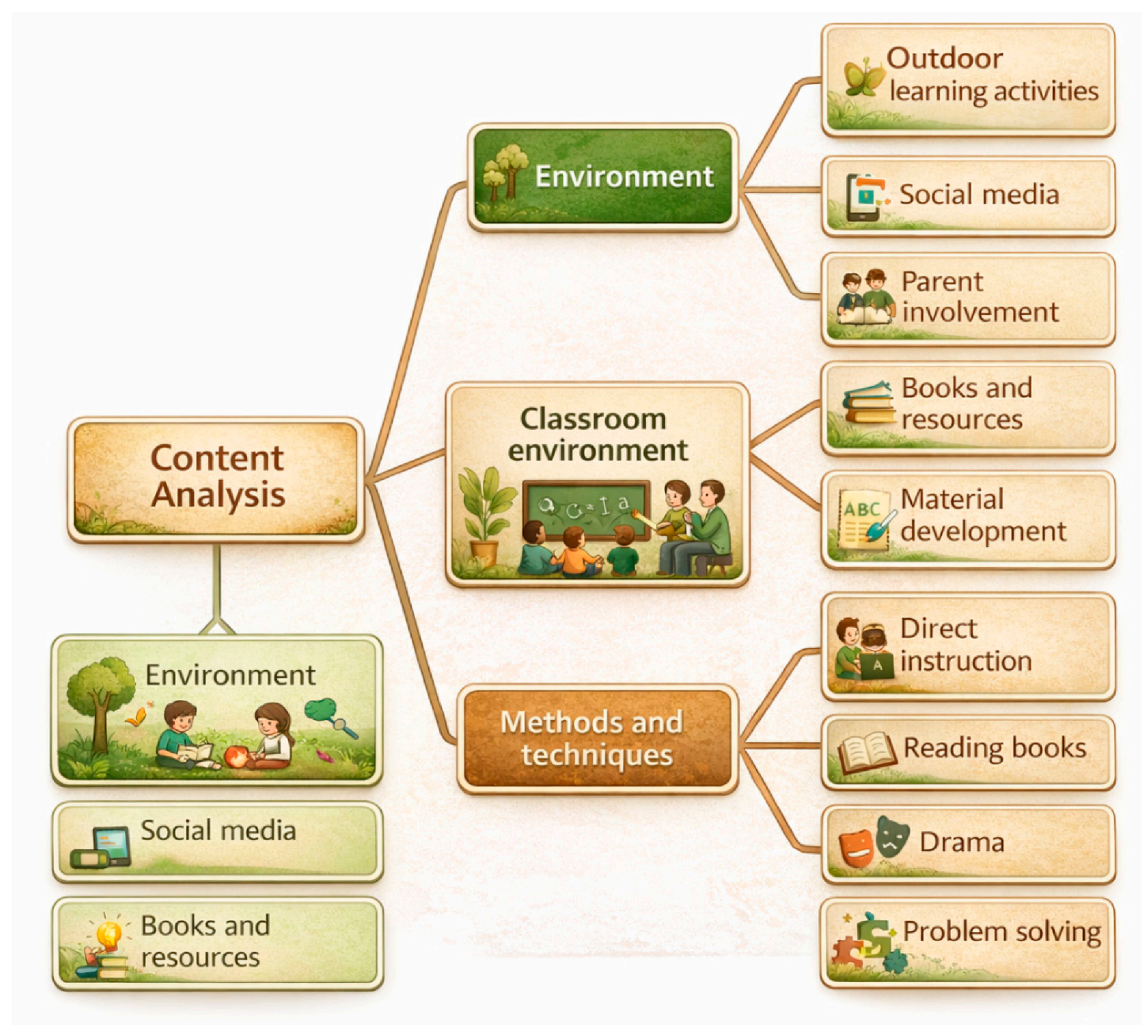
Content analysis.

As shown in Figure 3, the interview responses were coded according to the identified themes and subcategories. Under each theme, the relevant category, codes within that category, and the total repetition count of the codes are reflected in the table. For example, under the theme of “Classroom Environment,” there are categories such as “Material Development” and “Books and Other Resources,” and through related codes, the frequency of teachers’ opinions on these issues is demonstrated. Similarly, under the theme of “Environment,” subcategories like field trips, social media, and parental involvement are included, and the theme of “Methods and Techniques” is detailed with codes related to practices such as reading books, direct instruction, drama, and problem solving.

This figure enabled the systematic classification and analysis of teachers’ thoughts on early literacy practices and was utilized in formulating questions during the scale development process.

Determination of the measurement type: 5-point Likert-type.

Presentation for expert review: Data were collected from various sources for scale development and content validity, informed by data collected from semi-structured interviews with preschool teachers and structured observations of classroom and school physical conditions/materials pertinent to supporting early literacy. Children were not directly evaluated as part of this process; only the educational environment and early literacy resources available were observed. The items on the scale were restructured with the help of seven experts, such as academics and teachers. Expert opinions ensured content validity, which refers to how accurately a measurement tool represents its theoretical construct (Frank-Stromberg & Olsen, 2004). Academic experts evaluated the scale items for suitability, clarity, and content representation. While Lawshe’s (1975) methodology provides a framework for quantifying expert consensus, calculating the content validity ratio is not mandatory, and qualitative evaluations are accepted. Ayre and Scally (2014) advised against rigid interpretation of CVR values. Expert feedback was used qualitatively to refine scale items and enhance content coverage rather than using quantitative thresholds.

The preliminary pilot study: The scale items were administered face-to-face with nine teachers and then revised (such as shortening items that were not understood and correcting expression errors).

Pilot study: The scale was applied to 221 teachers. The study’s participants consisted of 221 preschool teachers working in public and private early childhood education institutions, with data collected both online and face-to-face. The scale was administered once for each teacher. The teachers did not respond regarding a specific child; instead, they reflected on their own general classroom practices and classroom discipline, as well as the practices and knowledge of early literacy development among their supporters. So, each teacher constituted one respondent and gave a single response set for their overall teaching practice. Participants were selected using purposive sampling methods. The inclusion criteria were possession of a valid preschool teaching diploma and at least one year of professional work experience. Participation was voluntary, and informed consent was obtained from all participants.

Examining the demographic characteristics of the teachers: Most participants were female (96.8%), with over half having 10+ years of teaching experience (56.9%). Classrooms predominantly had 11–20 students (65.6%). A total of 71.5% of teachers had regular reading habits. Student libraries existed in 55.2% of schools, while teacher libraries were present in 17.6%. Classroom libraries were present in 90.5% of rooms, and whiteboards in 88.7%. Teacher-owned books in classrooms were mostly 0–30 (76.5%), while student-owned books were commonly 30+ (43%). In total, 83.7% of teachers bought new books for students yearly, and 94.6% suggested book purchases. A total of 89.1% held regular reading hours. Student activities included author-signing days (25.8%), book fairs (14%), and library visits (43.4%). In total, 23.5% had library memberships, 18.1% had magazine subscriptions, and 17.6% had student magazine subscriptions. Regarding early literacy, 57% received training, 80.1% informed parents, and 78.7% followed social media updates. Learning centers with early literacy materials were available in 87.3% of schools.

Item analysis: Studies on content validity, construct validity, discriminant validity, external validity, and internal reliability were performed.

IBM SPSS Statistics version 25 and AMOS version 21 were used to analyze the research data. The dataset was examined for missing data, outliers, and normality, with necessary adjustments made. Normal distribution was examined using skewness and kurtosis. A multistage analysis process examined the scale’s psychometric properties. First, exploratory factor analysis (EFA) was conducted to determine structural characteristics and item relationships.

EFA explored the factor structure and examined item grouping dimensions. The dataset’s suitability for factor analysis was evaluated using the Kaiser–Meyer–Olkin (KMO) test and Bartlett’s Test of Sphericity. Confirmatory factor analysis (CFA) then tested the EFA model’s compatibility with theoretical structure, examining model relationships and item-factor fit. Various goodness-of-fit indices were considered to evaluate the model. For reliability assessment, Cronbach’s alpha coefficient, item-total correlations, and split-half reliability analyses were performed. Item discrimination analysis using 27% lower–upper group comparison and Hotelling’s T-squared test evaluated each item’s discrimination level. For scale validity, convergent and discriminant validity were assessed through Average Variance Extracted (AVE), Composite Reliability (CR), and HTMT ratios, evaluating subdimension distinctiveness and construct consistency.

### 2.2. How to Use the Ecological Theory-Based Early Literacy Assessment Scale-Teacher Form

The ETELAS-PT was created to evaluate preschool teachers’ practices and knowledge about promoting early literacy development using Bronfenbrenner’s ecological model. The instrument is completed by teachers to reflect general classroom practices and experiences rather than being specific to a child. The original questionnaire had items targeted at every ecological factor identified in the scale development. After the psychometric analyses, the final scale was made up of 25 Likert-type items divided into four subdimensions: the Mesosystem subdimension included nine items related to teachers’ instructional practices and interactions that connect children’s literacy experiences with the educational context. The Exosystem subdimension included 5 items related to practices of broader systems and resources that indirectly affect early literacy, including school-level interactions, as well as digital availability of support. The Macrosystem subdimension includes seven items that embody practices of teachers related to awareness and experiences in broader culture and society important to children’s literacy environments. The Chronosystem subdimension includes four items that assess teachers’ knowledge, monitoring, family guidance and support practices about children’s early literacy development across time. The individual indicators that fall within each subdimension are provided in the Supplementary Material. All items are rated on a 5-point Likert-type scale ranging from 0 (Never) to 4 (Always). It takes about 20 min to administer the instrument. The total instrument, organized by ecological dimensions, is available in the Supplementary Material.

To determine the construct validity of the ETELAS-PT, exploratory factor analysis (EFA) was conducted. The Kaiser–Meyer–Olkin (KMO) value was 0.894, indicating “excellent” data suitability for factor analysis. Bartlett’s Test of Sphericity was significant at χ^2^ = 3727.006; *p* < 0.001, confirming a suitable correlation structure. As shown in Table 1, EFA using principal component analysis (PCA) and varimax rotation revealed four distinct factors. The factor loadings ranged between 0.857 and 0.581 for the first factor, 0.768–0.651 for the second, 0.811–0.692 for the third, and 0.750–0.605 for the fourth factor. All items exceeded the 0.32 threshold suggested by Tabachnick and Fidell (2015), adequately representing their factors. The first, second, third, and fourth factors explained 22.488%, 16.591%, 13.099%, and 12.175% of the total variance, respectively, resulting in a total explained variance of 64.354%. Rather than interpreting the adequacy of the factor solution solely on the basis of a single variance threshold, the explained variance was considered together with the factor loadings, eigenvalues, and theoretical coherence of the four-factor structure. Accordingly, the four-factor solution was considered satisfactory for representing the multidimensional structure of the ETELAS-PT. The eigenvalues were 9.613, 3.253, 1.767, and 1.456 for the four factors respectively, all exceeding 1 (Alpar, 2013; Özdamar, 2017), indicating a meaningful structure.

**Figure 4:**
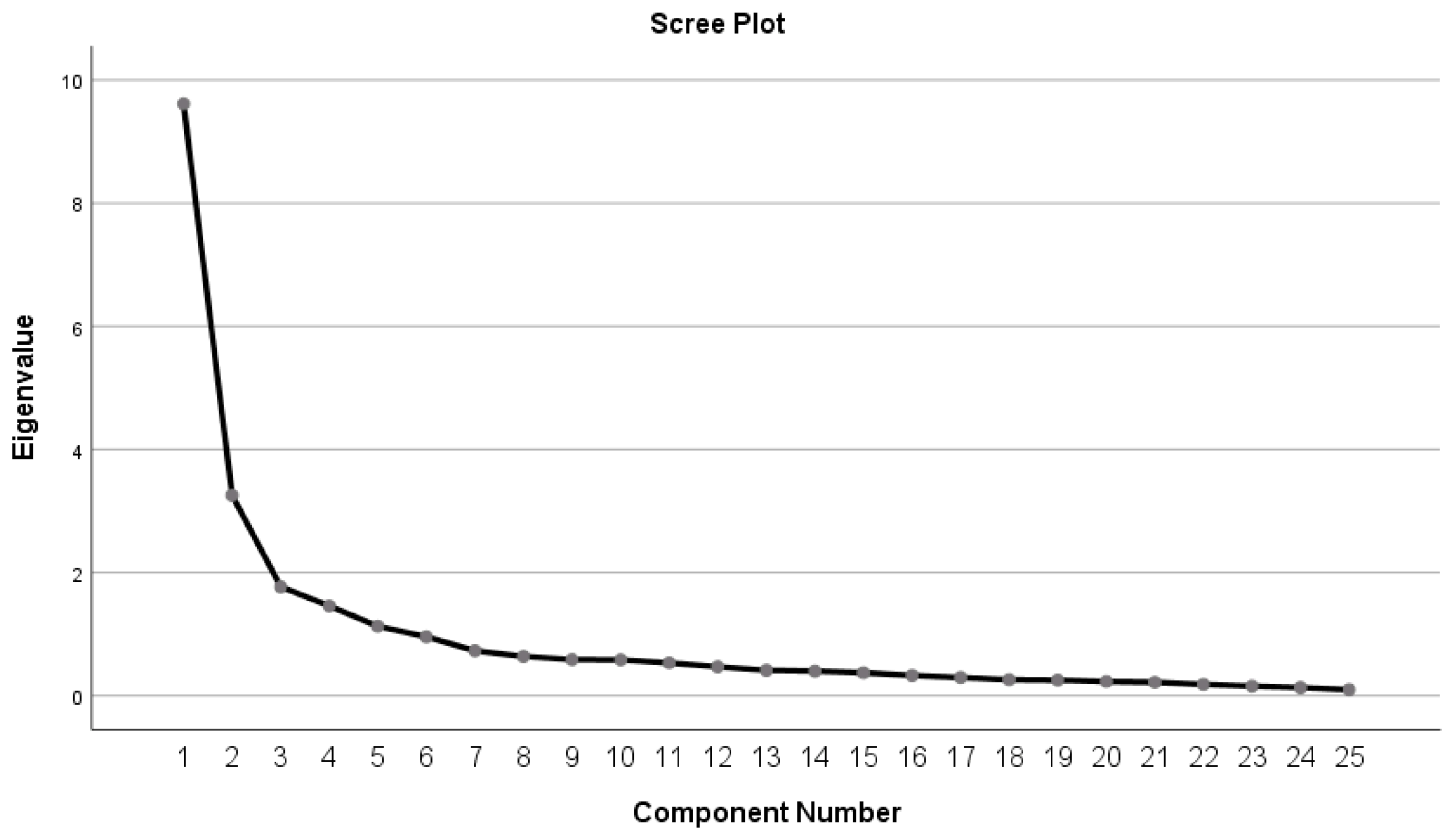
Scree plot.

The scree plot in Figure 4 shows the eigenvalue distribution from the factor analysis of the ETELAS-PT. The first component has a high eigenvalue (approximately 9.613), followed by a sharp drop in the second component (approximately three). From the third component onwards, eigenvalues decrease, with the curve becoming horizontal after the fourth component. Following Thompson (2008) and Alpar (2013)’s elbow method, this indicates four as the most appropriate number of factors, as eigenvalues become very close afterward and variance explained decreases significantly. From the fifth component, eigenvalues fall below 1, indicating no statistically significant information (Özdamar, 2017). Since only the first four factors exceed the eigenvalue threshold of 1 (Alpar, 2013; Özdamar, 2017), and considering both the Kaiser criterion and the scree plot’s elbow point, the scale’s four-factor structure is confirmed.

CR: The test value that examines the significance of the items; SE, on the other hand, is the standard error.

**Figure 5:**
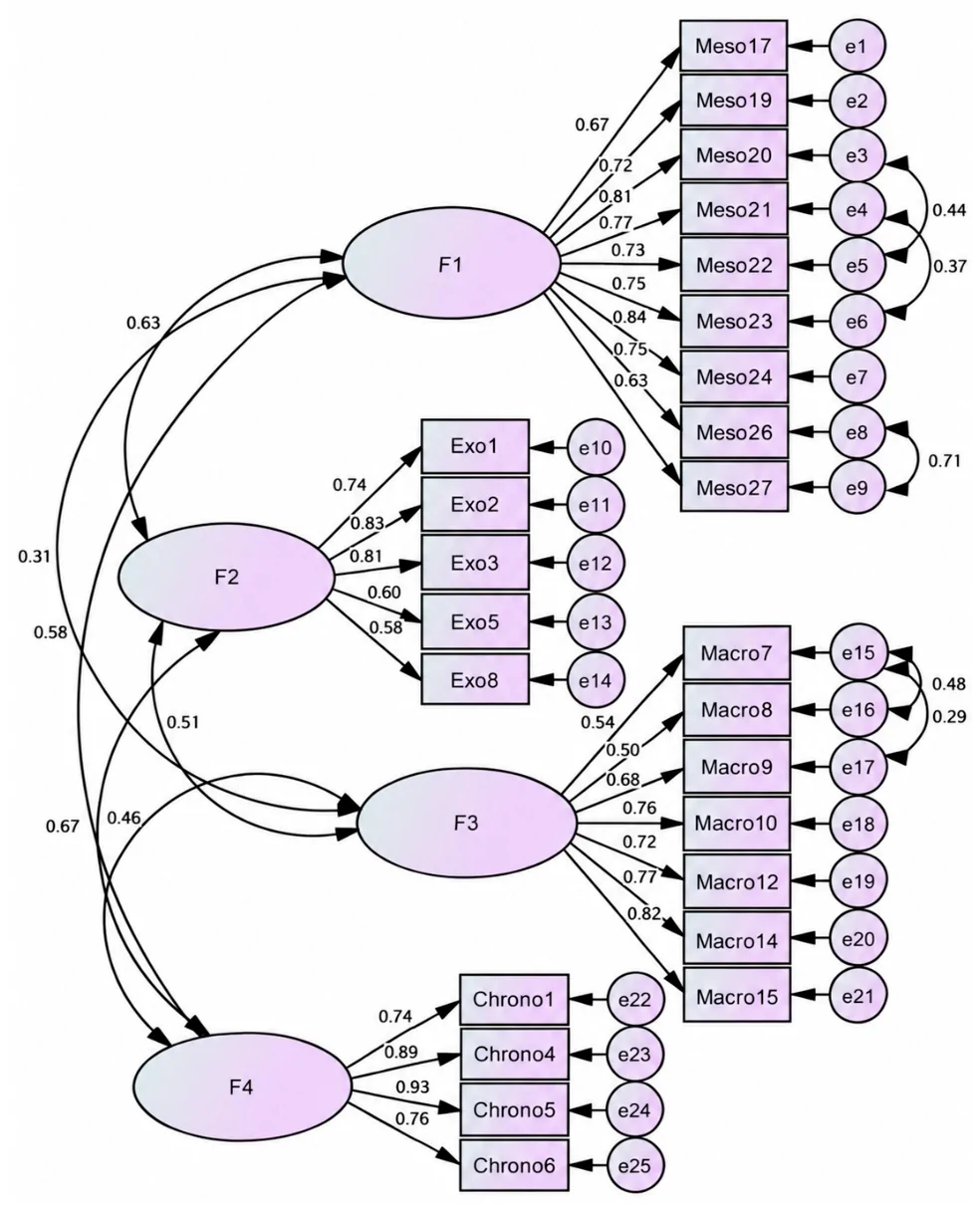
Results of the confirmatory factor analysis (standardized factor loadings). F1: Mesosystem, F2: Exosystem, F3: Macrosystem, F4: Chronosystem.

As shown in Figure 5, all standardized factor loadings were above 0.30. As presented in Table 2, all CR values were statistically significant (*p* < 0.001). According to Seçer (2018), factor loading values above 0.30 indicate that the items are suitable for the construct. The factor loadings of all items were relatively high.

The CFA (Table 3) results regarding the four-factor structure of the ETELAS-PT indicate that the model generally achieves an acceptable level of fit with the data. When examining the goodness-of-fit indices, the CMIN/df value was 2.241, which falls below the commonly accepted range of 3–5 in the literature, indicating an acceptable fit (Bentler, 1990). The standardized root mean square residual (SRMR) was 0.075, which is below 0.08, indicating that the model has an acceptable fit. Similarly, the RMSEA value of 0.069 is at the lower threshold of 0.08, indicating that the model demonstrates a reasonable level of fit (Hair et al., 2010). The absolute fit indices GFI (0.819) and AGFI (0.778) do not meet the criteria for excellent fit; however, the literature notes that these values may be lower in models with large sample sizes. Therefore, it can be stated that these two indices have values close to the acceptable limit. Considering that values of 0.90 and above are accepted as adequate fit for incremental fit indices in the literature (Hair et al., 2010; Marsh et al., 2004), the model largely meets these criteria.

The Table 4 presents the independent-group *t*-test results, showing the discriminative power of all items and item-total correlations. The item-total score correlation values and independent-samples *t*-test for discrimination were conducted across the entire scale for the awareness scale and for each subdimension of the adjustment scale. The minimum required value for the item-total test correlation to be considered adequate was specified as 0.30. Items with item correlation values below 0.30 were not included in the analysis. An examination of the item-total test correlation values for participants’ responses showed that there were no items below 0.30. As each subdimension was evaluated separately, the item-total correlation values for each subdimension were examined, and the correlation coefficients of the items were found to be significant. To determine the discriminative power of the items on the scale, the raw scores obtained from the scale were ranked from highest to lowest, and the mean scores of the groups in the lowest 27% and highest 27% were compared using an independent-samples *t*-test. The comparison revealed a statistically significant difference between the mean item scores in the lower and upper groups. Based on this, it can be said that the scale is discriminative in terms of measuring the desired quality of the content. In other words, when the 27% lower–upper group item analysis results for the measurement tool are examined, all items are discriminative.

Within the scope of reliability analysis, the internal consistency of the scale was evaluated using Cronbach’s alpha coefficient. A Cronbach’s alpha value in the range of 0.80–1.00 indicates that the scale has a high level of reliability (Tavşancıl, 2005). When examining the results presented in Table 5, the overall Cronbach’s alpha coefficient of the ETELAS-PT was 0.923, indicating that the scale as a whole possesses a high level of internal consistency. The reliability levels for the subdimensions of the scale were also quite high. The Cronbach’s alpha value calculated for the Mesosystem subdimension is 0.915, for the Exosystem subdimension 0.828, for the Macrosystem subdimension 0.866, and for the Chronosystem subdimension 0.893. In line with these findings, all subdimensions of the scale had an alpha coefficient above 0.80, indicating that all subdimensions also possessed a high level of reliability. Overall, both the full scale and all subscales confirmed a robust structure in terms of internal consistency, and it can be stated that the items included in the scale reliably measured the same conceptual construct.

### 2.3. Split-Half Reliability

The reliability of the scale was examined using a split-half test. This method is based on dividing a measurement tool into two equivalent parts and evaluating the relationship between the scores obtained from each part. If the measurement tool is reliable, the scores obtained from both halves are expected to be similar. The split-half test method can be applied by dividing the items odd–even or randomly (Baykul, 2015). In this study, the items were divided into two halves according to odd–even sequence. The Cronbach’s alpha coefficient was 0.859 for the first half and 0.836 for the second half. The correlation coefficient between the two halves was 0.923, indicating that the scale had a high level of internal consistency. Additionally, the Spearman–Brown correction coefficient was 0.960, and the Guttman Split-Half coefficient was 0.958. In the literature, a correlation above 0.70 between the two halves indicates that the measurement tool is reliable (Field, 2009). In this context, the findings revealed that the scale was highly reliable and valid.

## 3. Discussion

Traditional early literacy assessment practices in preschool settings have predominantly focused on individual child-level skills such as vocabulary, phonological awareness, and print knowledge, often relying on brief, screening-oriented instruments. Although these tools are effective in identifying children at risk for later reading difficulties, they provide limited insight into the broader environmental and sociocultural contexts that shape early literacy development. This narrow focus restricts teachers’ ability to form a holistic understanding of children’s literacy experiences and to design instruction that is responsive to contextual influences. To address this critical gap, the present study introduced and validated the Ecological Theory in Early Literacy Assessment: A Preschool Teacher-Focused Scale, grounded in Bronfenbrenner’s ecological systems theory, as a comprehensive teacher-focused assessment tool that systematically integrates environmental dimensions into early literacy evaluation.

### 3.1. Scale Development and Psychometric Findings

The development of the Ecological Theory in Early Literacy Assessment (A Preschool Teacher-Focused Scale) followed a structured, theory-driven process. Items were generated based on an extensive review of Bronfenbrenner’s ecological systems theory and contemporary early literacy frameworks, and were mapped onto five ecological subdimensions: microsystem, mesosystem, exosystem, macrosystem, and chronosystem. Content validity was established through expert review involving early childhood education academics and experienced preschool teachers, and revisions were made to enhance clarity and representativeness. The scale was subsequently pilot-tested and validated using data collected from preschool teachers working in diverse educational settings.

Psychometric properties were examined through internal-consistency reliability analyses as well as exploratory and confirmatory factor analyses. Reliability findings indicated high internal consistency for both the total scale and its subdimensions. The Cronbach’s alpha coefficient for the total scale was α = 0.923, demonstrating excellent reliability. Subdimension reliability coefficients were also high, with α = 0.915 for the mesosystem, α = 0.828 for the exosystem, α = 0.866 for the macrosystem, and α = 0.893 for the chronosystem. All coefficients exceeded the commonly accepted threshold of 0.70, indicating that the scale consistently measures its intended constructs.

### 3.2. Exploratory and Confirmatory Factor Structure

Construct validity was first examined through exploratory factor analysis (EFA). The suitability of the data for factor analysis was confirmed by a Kaiser–Meyer–Olkin (KMO) value of 0.894, classified as “excellent” (Alpar, 2013), and a significant Bartlett’s Test of Sphericity (χ^2^ = 3727.006, *p* < 0.001). EFA conducted using principal component analysis with varimax rotation revealed a four-factor structure, consistent with the ecological framework underlying the scale.

Factor loadings ranged from 0.581 to 0.857 for the first factor, 0.651 to 0.768 for the second factor, 0.692 to 0.811 for the third factor, and 0.605 to 0.750 for the fourth factor. All loadings exceeded the 0.32 criterion suggested by Tabachnick and Fidell (2015), indicating that each item adequately represented its respective factor. The four factors accounted for 64.354% of the total variance, with individual contributions of 22.488%, 16.591%, 13.099%, and 12.175%, respectively. Eigenvalues for the four factors (9.613, 3.253, 1.767, and 1.456) all exceeded the Kaiser criterion of one, supporting the retention of a four-factor solution. The scree plot further confirmed this structure, showing a clear elbow point after the fourth factor, consistent with the elbow method (Thompson, 2008).

Following EFA, confirmatory factor analysis (CFA) was conducted to test the four-factor model. The CFA results indicated an acceptable level of model fit. The CMIN/df ratio was 2.241, indicating an acceptable model fit. The remaining fit indices were evaluated using established recommendations for structural equation modeling (Kline, 2023). The SRMR value of 0.075 and RMSEA value of 0.069 were both within acceptable ranges, indicating satisfactory residual and approximate fit (Hair et al., 2010). Although the absolute fit indices GFI (0.819) and AGFI (0.778) did not reach the criteria for excellent fit, these indices are known to be sensitive to sample size and model complexity. Incremental fit indices largely met recommended thresholds, and taken together, the CFA results support the adequacy of the proposed four-factor structure.

### 3.3. Interpretation of Findings in Relation to Existing Assessment Practices

The results of the exploratory factor analysis (EFA) are consistent with those of the confirmatory factor analysis (CFA), thus increasing confidence in ETELAS-PT construct validity. These create a four-factor structure, identified as Mesosystem, Exosystem, Macrosystem and Chronosystem; each of which shows separate but connected ecological aspects to preschool teachers’ attitudes/beliefs regarding their practices related to helping preschool children learn about literacy. The item-development process involved all the levels of Bronfenbrenner’s ecological framework (Microsystem) when developing the ETELAS-PT; however, ultimately 25 Likert-type items covering four subdimensions formed the psychometrically validated scale. Among the Microsystem items of the initial assessment framework, only those that did not load took on a separate facet role in the final scale structure.

The four dimensions retained go beyond direct classroom activities, assessing the interactions that teachers have in the educational context, the resources and technology used (isolating institutional level and digital level), cultural experiences and societal incorporation of practices, monitoring over time as well as supporting families. In this regard, the ETELAS-PT offers an ecological perspective on teachers, which adds a necessary level not covered by literacy assessments focused solely at the child-level.

### 3.4. Comparison with Screening and Curriculum-Based Assessment Tools

Early literacy screening tools like IGDIs, EL-IGDIs, and FastBridge serve as quick indicators of children’s language and reading capabilities to identify at-risk children (Missall et al., 2006; Wackerle-Hollman et al., 2018). Yet, these tools emphasize child-level performance and are not intended to systematically evaluate the practices and knowledge of teachers in the wider ecological contexts that underpin early literacy. In response to recommendations that early childhood assessment systems are best positioned to guide instructional decision-making rather than simply screen (McConnell et al., 2014), the ETELAS-PT takes a complementary perspective by examining how preschool teachers support early literacy across ecological domains.

Research on curriculum-based measurement also underscores the need for assessment systems that yield meaningful information to guide educational decision-making (Buysse & Peisner-Feinberg, 2013; Greenwood et al., 2013). Unlike screening and curriculum-based measures that are focused on children, the ETELAS-PT does not conduct performance assessments of children’s literacy or diagnose literacy difficulties. Rather, it allows teachers to systematically reflect on their own practices and awareness of the Mesosystem, Exosystem, Macrosystem, and Chronosystem. Therefore, we designed the ETELAS-PT as a more eco-informative supplement to child-level assessment tools in early literacy assessment and instructional preparation.

### 3.5. Ecological Variables, Teacher Practices, and Implications

Studies have demonstrated that early literacy outcomes are significantly associated with age, sociodemographic characteristics, and environmental contexts (Ecalle et al., 2020). Children’s self-regulation and executive functioning observed by teachers during literacy activities play a critical role in literacy development (Lonigan et al., 2011). The chronosystem dimension allows teachers to consider developmental changes and environmental influences over time, thereby supporting longitudinal assessment practices. Teachers’ beliefs and perspectives regarding early literacy can influence how literacy goals and practices are conceptualized in preschool settings (Powell et al., 2008) and professional development enhances teachers’ assessment competence (Cunningham et al., 2009). Thus, the ETELAS-PT can not only serve as an assessment tool but also a reflection tool for preschool teachers to help them identify which areas of their early literacy practices might need additional attention or support. At the classroom level, teachers can compare their responses across the four ecological dimensions throughout the year to examine frequency and depth of literacy-supporting practices, breadth of educational and digital resources used, inclusion of cultural and societal experiences, and use of monitoring and family-support practices over time. Some dimensions receive lower scores while others can be relatively high; these ratings may suggest strategies for improvement, such as seeking professional development, instructional planning or environmental support. At the institutional level, aggregated outcomes may also shed light on shared professional support needs of preschool teachers, thereby providing important input for tailored professional development planning. Significantly, the ETELAS-PT is designed to inform teachers’ reflection and awareness of practices rather than be a tool to diagnose literacy strengths of particular children.

## 4. Conclusions

In conclusion, the ETELAS-PT is a theoretically anchored and psychometrically sound measure of preschool teachers’ practices regarding supporting literacy development in their students as well as their awareness of factors included in Bronfenbrenner’s ecological model. Organized into four dimensional states—Mesosystem, Exosystem, Macrosystem, and Chronosystem—it supplies a practical ecological view of early literacy assessment from the teacher level rather than the child level. The ETELAS-PT provides preschool teachers with an opportunity to identify the early literacy-supporting practices they employ, based on reflection and observation rather than assessment or diagnosis of young children’s literacy abilities. The instrument might assist teachers in determining which areas deserve greater attention when it comes to planning for instruction, using instructional and digital resources, integrating cultural and societal experiences into instruction, observing the early literacy development of their students, and engaging families in support. At the institutional level, overall scores could also identify professional development needs and assist with planning need-based support for preschool teachers. Therefore, the ETELAS-PT may fill gaps in current early literacy assessment tools targeted at children by providing an ecological, teacher-centered view into helping children with early literacy support and planning for educational needs.

## Figures and Tables

**Table 1:** Results of the exploratory factor analysis.

Sub Scale	Factors			
	Factor 1	Factor 2	Factor 3	Factor 4
Meso21	0.857			
Meso20	0.816			
Meso22	0.799			
Meso23	0.782			
Meso24	0.766			
Meso26	0.757			
Meso17	0.653			
Meso19	0.604			
Meso27	0.581			
Macro15		0.768		
Macro10		0.754		
Macro9		0.730		
Macro14		0.730		
Macro7		0.705		
Macro12		0.676		
Macro8		0.651		
Chrono5			0.811	
Chrono4			0.803	
Chrono1			0.797	
Chrono6			0.692	
Exo5				0.750
Exo2				0.655
Exo8				0.650
Exo1				0.639
Exo3				0.605
Eigenvalue	9.613	3.253	1.767	1.456
Variance	22.488	16.591	13.099	12.175

KMO and Bartlett’s Test → KMO = 0.894; Bartlett’s Test (df) = 3727.006 (300); *p* = 0.000; total explained variance = 64.354.

**Table 2:** Factor loadings of the CFA (confirmatory factor analysis).

	Standardized Factor Loading	Unstandardized Factor Loadings	Standard Error	Critical Ratio	*p*
Meso17	0.667	1.000			<0.001
Meso19	0.724	1.105	0.116	9.563	<0.001
Meso20	0.815	1.041	0.099	10.559	<0.001
Meso21	0.771	0.843	0.084	10.076	<0.001
Meso22	0.730	0.872	0.091	9.602	<0.001
Meso23	0.753	0.709	0.072	9.868	<0.001
Meso24	0.836	1.017	0.094	10.787	<0.001
Meso26	0.755	0.805	0.081	9.915	<0.001
Meso27	0.629	0.614	0.073	8.427	<0.001
Exo1	0.738	1.000			<0.001
Exo2	0.826	1.111	0.095	11.719	<0.001
Exo3	0.812	1.056	0.091	11.544	<0.001
Exo5	0.603	0.905	0.106	8.532	<0.001
Exo8	0.584	1.044	0.126	8.258	<0.001
Macro7	0.542	1.000			<0.001
Macro8	0.504	0.900	0.109	8.255	<0.001
Macro9	0.685	1.294	0.150	8.614	<0.001
Macro10	0.761	0.954	0.123	7.751	<0.001
Macro12	0.718	1.225	0.163	7.512	<0.001
Macro14	0.772	1.663	0.213	7.810	<0.001
Macro15	0.824	1.563	0.194	8.052	<0.001
Chrono1	0.739	1.000			<0.001
Chrono4	0.888	1.272	0.095	13.420	<0.001
Chrono5	0.928	1.518	0.109	13.941	<0.001
Chrono6	0.756	1.263	0.112	11.274	<0.001

**Table 3:** Goodness-of-fit indices of the CFA (confirmatory factor analysis).

Index	Excellent Fit	Acceptable Fit	Model Compliance
CMIN/df	≤5	≤3	2.241
RMSEA	≤0.08	≤0.05	0.069
SRMR	≤0.08	≤0.05	0.075
TLI	≥0.90	≥0.95	0.896
CFI	≥0.90	≥0.95	0.909
IFI	≥0.90	≥0.95	0.910

**Table 4:** Item analysis. * *p* < 0.001, ** *p* < 0.05.

Item	Group	Mean	SD	r	Cronbach’s Alpha if Item Deleted	t (** Upper–Lower 27%)	*p* (** Upper–Lower 27%)
**ETELAS-PT (Mesosystem)**							
Meso17	Upper	4.00	0.00	0.634	0.913	8.858	<0.001 *
	Lower	2.55	1.27				
Meso19	Upper	4.00	0.00	0.672	0.911	11.412	<0.001 *
	Lower	2.25	1.19				
Meso20	Upper	4.00	0.00	0.801	0.899	12.034	<0.001 *
	Lower	2.50	0.97				
Meso21	Upper	4.00	0.00	0.761	0.902	7.215	<0.001 *
	Lower	3.00	1.07				
Meso22	Upper	4.00	0.00	0.709	0.905	11.782	<0.001 *
	Lower	2.67	0.88				
Meso23	Upper	4.00	0.00	0.726	0.906	7.371	<0.001 *
	Lower	3.13	0.91				
Meso24	Upper	4.00	0.00	0.787	0.900	9.842	<0.001 *
	Lower	2.72	1.01				
Meso26	Upper	4.00	0.00	0.745	0.904	7.019	<0.001 *
	Lower	3.05	1.05				
Meso27	Upper	4.00	0.00	0.615	0.912	7.225	<0.001 *
	Lower	3.13	0.93				
**ETELAS-PT (Exosystem)**							
Exo 1	Upper	3.93	0.31	0.645	0.790	11.931	<0.001 *
	Lower	1.93	1.26				
Exo 2	Upper	3.88	0.32	0.716	0.771	16.051	<0.001 *
	Lower	1.67	1.02				
Exo 3	Upper	3.95	0.29	0.709	0.775	15.365	<0.001 *
	Lower	1.83	1.03				
Exo 5	Upper	3.82	0.39	0.565	0.812	13.497	<0.001 *
	Lower	1.55	1.24				
Exo 8	Upper	3.68	0.62	0.551	0.827	17.926	<0.001 *
	Lower	0.77	1.09				
**ETELAS-PT (Macrosystem)**							
Macro 7	Upper	3.95	0.22	0.613	0.851	13.133	<0.001 *
	Lower	1.90	1.19				
Macro 8	Upper	3.98	0.13	0.561	0.858	10.244	<0.001 *
	Lower	2.30	1.27				
Macro 9	Upper	3.97	0.18	0.692	0.840	13.254	<0.001 *
	Lower	1.87	1.21				
Macro 10	Upper	4.00	0.00	0.688	0.848	9.575	<0.001 *
	Lower	2.80	0.97				
Macro 12	Upper	3.98	0.13	0.617	0.851	9.990	<0.001 *
	Lower	2.35	1.26				
Macro 14	Upper	3.93	0.25	0.661	0.846	18.614	<0.001 *
	Lower	1.33	1.05				
Macro15	Upper	4.00	0.00	0.705	0.838	15.642	<0.001 *
	Lower	1.75	1.11				
**ETELAS-PT (Chronosystem)**							
Chrono 1	Upper	4.00	0.00	0.702	0.885	7.140	<0.001 *
	Lower	3.12	0.96				
Chrono 4	Upper	4.00	0.00	0.819	0.844	14.922	<0.001 *
	Lower	2.72	0.67				
Chrono 5	Upper	4.00	0.00	0.848	0.829	12.501	<0.001 *
	Lower	2.60	0.87				
Chrono 6	Upper	4.00	0.00	0.709	0.886	12.672	<0.001 *
	Lower	2.62	0.85				

**Table 5:** Reliability results.

Scale and Subdimensions	Cronbach’s α	McDonald’s ω
ETELAS-PT	0.923	0.932
ETELAS-PT (Mesosystem)	0.915	0.922
ETELAS-PT (Exosystem)	0.828	0.842
ETELAS-PT (Macrosystem)	0.866	0.873
ETELAS-PT (Chronosystem)	0.893	0.898

## Data Availability

The data supporting the findings of this study are available from the corresponding author upon reasonable request. The data are not publicly available due to privacy and ethical restrictions.

## Supplementary Materials

The following supporting information can be downloaded at: https://www.mdpi.com/article/10.3390/bs16101777/s1, Supplementary Material S1: ETELAS-PT—A Preschool Teacher-Focused Scale.
