# Supplementary material for: Ecological Theory in Early Literacy Assessment: A Preschool Teacher-Focused Scale (ETELAS-PT)
Source: Behav Sci (Basel). 2026 Sep 29;16(10):1777. doi: 10.3390/bs16101777 (PMC13701341; doi:10.3390/bs16101777)
Supplement: Supplementary file 1 [file behavsci-16-01777-s001.zip › behavsci-4380347-supplementary.pdf]

Dear Teacher,

This scale has been developed to gain a better understanding of the school environment and school-based practices that support your students' early literacy development. It consists of sections designed to assess your practices for supporting early literacy within the framework of Bronfenbrenner's Ecological Theory. The scale takes approximately 20 minutes to complete and includes both yes/no questions and rating-scale items. Beginning with Section II-b, items are rated on a Likert-type scale (Never-Rarely-Sometimes-Often-Always).

Section I

Personal Information of the Teacher

Gender:

Year of birth / Age:

High school graduated from:

University and department graduated from:

Year of graduation:

Professional Information

How many years have you been working as a preschool teacher?

Type of institution you work at: Public ( ) Private ( ) Foundation ( ) Other ( )

Province/District where you work:

How many students are in your class?

Age group in your class:

Section II Ecological Theory in Early Literacy Assessment: A Preschool Teacher-Focused Scale (ETELAS-PT)

**Section II-a: Ecological Theory in Early Literacy Assessment: A Preschool Teacher-Focused Scale (ETELAS-PT)Microsystem)**<br>In the ecologically-based early literacy approach, the physical environment that students are in plays a major role in their learning processes. You can make the environments encountered at school more suitable for learning to support your students' early literacy skills.			
**Assessment Criteria**	**No**	**Yes**	**If yes, could you explain?**
Do you have a regular reading habit?			
If yes, could you specify how many days a week and at what times you do this?			
Is there a library at your school that serves your students?			
Is there a library at your school that serves the teachers?			
Is there a bookshelf in your classroom?			
Is there a writing board (e.g., a wipeable board or similar area) in your classroom?			
Could you tell us the number of books you have in your classroom?			
Could you tell us the number of books belonging to your students in your classroom?			
Do you buy new books for your students throughout the year?			
Do you make suggestions for buying books?			
Do you regularly have reading hours with your students? If yes, how often?			
Have you attended an author's signing event with your students?			
Have you attended a book fair with your students?			
Have you organized a library visit with your students?			
Do you have an active library membership?			
Are you regularly subscribed to any magazine?			
Are you subscribed to a specific magazine for your students?			

Assessment Criteria	No	Yes	If yes, could you explain?
Early literacy refers to the skills that children develop before they learn to read and write. These skills include children's language development, vocabulary, ability to recognize sounds, and their relationships with books in the preschool period. Early literacy forms the foundation for children's reading and writing processes in primary school. As a teacher, have you received training on topics like Early Literacy/Readiness for Reading?			
Do you provide information to parents about early literacy?			
Do you follow pages or individuals on social media that support early literacy skills?			
Do you purchase books/toys for your classroom to support early literacy skills? (Counting, matching pictures, mazes, puzzles, etc.)			
Are there toys/materials in learning centers that support early literacy?			

Section II-b: Ecological Theory in Early Literacy Assessment: A Preschool Teacher-Focused Scale (Mesosystem)

The mesosystem aspect of ecologically based early literacy assessment scale focuses on the interaction of environmental factors that influence students' development (e.g., school-family collaboration, teacher-parent communication, and teacher-peer relationships). This dimension aims to assess the collaboration teachers establish with families and other educational stakeholders and how this collaboration contributes to students' early literacy development.

**Assessment Criteria**	**Never (0)**	**Rarely (1)**	**Sometimes (2)**	**Often (3)**	**Always (4)**
Before reading a storybook to my students, I ask them questions to guess the title.					
While reading a storybook to my students, I ask them to name an object or situation by pointing to it.					
I ask my students to retell the story (in their own words).					
After reading a storybook to my students, I ask them questions about the story. (For example, the 5W1H questions: What, where, when, how, why, and who)					
After reading a storybook to my students, I asked them to recount the story.					
After reading a storybook to my students, I give them the opportunity to ask questions about the story.					
I show my students pictures from the storybook and ask them to describe them.					
In my activities, I show my students the numbers.					
In my activities, I show my students the shapes.					

Section II-c: Ecological Theory Based Early Literacy Assessment Scale (Exosystem)

The exosystem dimension of ecological-based early literacy assessment scale focuses on environmental factors in which the student is not directly involved but that indirectly affect their development (such as educational policies, media, digital resources, and parents' working conditions).

Assessment Criteria	Never (0)	Rarely (1)	Sometimes (2)	Often (3)	Always (4)
I use media channels (websites, YouTube channels, apps, etc.) to support my students' early literacy skills.					
I offer suggestions for developing early literacy skills in school administration.					
I exchanged ideas with classroom teachers about preparing for reading.					
I use artificial intelligence to improve my early-literacy skills.					
I use our language with subtitles when showing videos or similar documentaries as part of my training programme.					

Section II-d: Ecological Theory Based Early Literacy Assessment Scale (Macrosystem)

The macrosystem dimension of ecologically based early literacy assessment scale encompasses broader social, cultural, and economic factors. These elements are environmental layers through which external factors, such as educational policies, cultural norms, economic status, and media, indirectly influence early literacy development. The macrosystem aims to understand how teachers remain sensitive to changes within this wider social context and how they consider these factors to support their students' literacy development

Assessment Criteria	Never (0)	Rarely (1)	Sometimes (2)	Often (3)	Always (4)
I teach my students about their home address.					
I ask my students to learn their parents' mobile phone numbers.					
I show my students the amounts of money used in daily life (e.g., 10 TL).					
I show my students a map of our country.					
I examined world maps with my students.					

Assessment Criteria	Never (0)	Rarely (1)	Sometimes (2)	Often (3)	Always (4)
I provide information on the currencies of different countries.					
I provide information on flags of different countries.					

Section II-e: Ecological Theory Based Early Literacy Assessment Scale (Chronosystem)

The ecologically based early literacy assessment scale chronosystem dimension refers to environmental factors that change over time and periods in an individual's life. This level includes historical, social, and personal life events that influence students' development. In educational processes, dynamics such as socio-economic changes experienced by the student, transformations within the family structure, developments in educational policies, and societal trends are considered, creating long-term effects on literacy skills.

Assessment Criteria	Never (0)	Rarely (1)	Sometimes (2)	Often (3)	Always (4)
I am aware of the impact of early literacy skills on the development of my students.					
I communicate with the families of students whom I consider at risk in terms of early literacy skills, and I provide guidance to ensure they are supported at home as well.					
I inform families about how to support early literacy skills at home.					
I monitor my students' progress in the area of early literacy skills through their portfolio files.					
